# Thermal Tolerance of *Crassostrea* (*Magallana*) *ariakensis* to Nuclear Plant Warm Water Discharges

**DOI:** 10.3390/biology14030311

**Published:** 2025-03-19

**Authors:** Lei Li, Longyu Liu, Cong Yan, Liang Wang, Yuanlv Ye, Lu Chen, Xiong Zou, Haijing Zhang, Mengni Zeng, Mei Jiang

**Affiliations:** 1 East China Sea Fisheries Research Institute, Shanghai 200090, China; zheyilee@126.com (L.L.); liulongyu1219@163.com (L.L.); zouyxiang9901@163.com (X.Z.); mengnizeng@163.com (M.Z.); 2 College of Marine Science and Environment Engineering, Dalian Ocean University, Dalian 116023, China; 3 Yancheng Institute of Technology, College of Marine and Biological Engineering, Yancheng 224051, China; 19857293926@163.com; 4 State Environmental Protection Key Laboratory of Nuclear and Radiation Safety Regulatory Simulation and Validation, Nuclear and Radiation Safety Center, Ministry of Ecology and Environment, Beijing 100082, China; wangli-ang@chinansc.cn (L.W.); yeyuanlv@chinansc.cn (Y.Y.); chenlu@chinansc.cn (L.C.); 5 East China Sea Survey Center, Ministry of Natural Resources, Shanghai 200137, China; hjzhang202401@163.com

**Keywords:** thermal tolerance, *Crassostrea (Magallana) ariakensis*, nuclear plants, warm water discharges

## Abstract

Nuclear power plants utilize great quantities of seawater to cool down, resulting in substantial warm water discharges that may affect nearby fisheries and marine ecosystems. This study focused on *Crassostrea* (*Magallana*) *ariakensis*, a commercially farmed oyster species along the southern coast of China. To evaluate the thermal impacts of warm water discharges from nuclear power plants, indoor simulations replicated seasonal water temperature conditions near coastal facilities (26 °C in spring and autumn, 16 °C in winter, and 30 °C in summer). We conducted thermal tolerance static and dynamic experiments, along with a 51-day long-term experiment on suitable growth under different acclimation temperatures. The thermal effects of warm water discharges on *C. ariakensis* were systematically assessed through survival, growth, digestibility, and nutritional quality. The results showed that the discomfort temperature range of *C. ariakensis* was (48.6 ± 1.2)~(58.9 ± 3.0) °C, the critical thermal maxima (CTM) value range of *C. ariakensis* was (51.6 ± 1.4)~(61.2 ± 2.2) °C, and the incipient lethal temperature (ILT_50_) of *C. ariakensis* was 45.61 °C, 53.71 °C, and 55.90 °C, respectively; all these values increased gradually with the rise of acclimation temperature. After the 51-day long-term experiment on suitable growth, the temperature increase of 1 °C, 2 °C and 4 °C did not affect the soft tissue wet weight, condition index, moisture content, and fat content of *C. ariakensis*, but the amylase activity in digestive gland tissue decreased in different temperature experimental groups. The experimental results show that the influence of temperature rise on the growth and physiological metabolism of *C. ariakensis* is limited. However, based on the normal habitat temperature in summer, the long-term effects of temperature rise caused by warm water discharges need to be paid attention to.

## Simple Summary

Nuclear power, recognized for its clean and cost-effective energy production, relies heavily on seawater for cooling, resulting in significant warm water discharges. These discharges can raise the temperature of receiving marine environments and harm local fisheries and ecosystems. This study aims to evaluate the thermal tolerance of *Crassostrea* (*Magallana*) *ariakensis* farmed along the southern coast of China and to provide essential data and technical support for the optimization of thermal discharge management in nuclear power plants and the rational layout of marine aquaculture. In this study, by simulating the water temperature environments in different seasons near the coastal nuclear power plants, we carried out a series of thermal tolerance experiments of *C. ariakensis*. The results reveal that the influence of temperature rise on the growth and physiological metabolism of *C. ariakensis* is limited; however, the long-term consequences should raise concerns given the typical summer habitat temperature.

## 1. Introduction

Nuclear power, recognized as a safe, clean, and cost-effective energy source, significantly contributes to CO_2_ emissions reduction and global climate change mitigation [1]. According to the National Nuclear Safety Administration, as of March 2022, mainland China had 71 nuclear power plants either under construction or in operation, all situated along the coast. Projections indicate that China’s nuclear power sector will experience rapid growth, accounting for 15–22% of the country’s electricity generation between 2030 and 2050 [2].

The operation of nuclear power plants generates substantial heat, requiring significant amounts of cooling water. Most of China’s nuclear power plants use a once-through cooling system, where water is drawn in, circulated for cooling, and then released back into the environment. Approximately three-quarters of the heat generated by nuclear reactions is dissipated into the seawater used as a coolant. Research shows that the temperature of the discharged water can be 7–10 °C higher than the intake water [3]. Furthermore, this thermal discharge may contain chlorides and other antifouling agents, potentially leading to cumulative effects. The physiological processes and behaviors of marine species and ecosystems are influenced by temperature, a crucial environmental component that can change geographic distribution, community structure, and ecosystem dynamics [4]. Consequently, thermal discharge may have significant impacts on the organisms and ecosystems of the receiving water bodies [5,6]. This thermal effect shares some similarities with the ocean warming caused by global climate change [4].

Given the potential risks of thermal discharge from nuclear power plants to marine ecosystems, its impact on marine species and habitats has become a key consideration in the design and selection of nuclear power plant sites [7]. Since China’s aquaculture production has long been the world’s largest, and there are numerous farming activities along the coast, the impact of warm water discharges on aquaculture species has always been a crucial factor in the planning and constructing of nuclear power plants in China. Shellfish dominate marine aquaculture in China in terms of both area and production, with oysters being the most widely farmed species. These farming areas are typically located in nearshore waters [8].

*Crassostrea* (*Magallana*) *ariakensis*, commonly known as the Suminoe oyster, belongs to the class Bivalvia, order Ostreida, family Ostreidae, and genus Crassostrea. It is an economically important shellfish species extensively farmed along the southern coast of China. The Suminoe oyster exhibits moderate thermal tolerance; however, this tolerance range has a limit, and temperatures above this can negatively impact its growth rate, reproductive success, and seedling survival [9]. However, current research primarily focuses on the oyster’s short-term tolerance to high temperatures [9], with limited studies on the effects of seawater temperature rise under actual nuclear power plant operating conditions.

This study investigates the thermal tolerance of the Suminoe oyster *C. ariakensis* by simulating the thermal discharge conditions of coastal nuclear power plants during various seasons. The research question is how rising temperatures affect the survival, growth, nutritional quality, and digestive capacity of *C. ariakensis*. The findings can provide critical data and technical support for optimizing thermal discharge management in nuclear power plants and for strategic planning in marine aquaculture.

## 2. Materials and Methods

### 2.1. Experimental Materials

The oysters (gross weight: 61.7 ± 10.1 g, soft tissue wet weight: 4.1 ± 1.8 g) were collected from the oyster farming rafts in Hongsha Bay, Fangchenggang City, Guangxi Province, which is located approximately 15 km from the nearest nuclear power plant. Specifically, the oyster farming rafts are approximately 11 km away from the nuclear plant’s 1 °C thermal discharge envelope, which is the area where water temperatures increased by 1 °C due to the plant operation [10]. Since the sampling site lies outside this envelope, the oysters in this area were not affected by thermal discharge. The average seawater temperature when the oysters were harvested was 23 °C.

### 2.2. Experimental Methods

#### 2.2.1. Acclimation

##### Thermal Tolerance Experiment

The experiment was conducted at a marine aquaculture facility in Sanniang Bay, Qinzhou City, Guangxi Zhuang Autonomous Region. The oysters were initially cleaned to remove epibionts and then temporarily retained in polyethylene containers. Based on surface seawater temperature data from a coastal nuclear power plant in Guangxi from 2015 to 2019 (average in spring: 25.0 ± 0.7 °C, in summer: 30.2 ± 0.3 °C, in autumn: 26.5 ± 0.4 °C, and in winter: 16.6 ± 0.6 °C), three acclimation temperatures were set: 16 °C (average water temperature in winter), 26 °C (average water temperature in spring and autumn), and 30 °C (average water temperature in summer). Acclimation was carried out in plastic tanks (150 L) at the rate of 1 °C/d from laboratory temperature (23 °C) to reach experimental temperatures (16, 26, and 30 °C). Once the target temperature was reached, the oysters were acclimated for an additional 5 days. The 26 °C and 30 °C acclimation temperatures were controlled using heating rods (±0.5 °C), while the 16 °C temperature was controlled using a chiller, and the water temperature was monitored with a thermometer (accuracy 0.1 °C). The experimental water, sourced from natural seawater (pH 8.1, salinity 26‰) after filtration and precipitation, was maintained with an aeration pump to ensure dissolved oxygen levels above 6.0 mg/L, under a natural photoperiod of 12 h light: 12 h dark. During acclimation, the oysters were fed twice daily (at 09:00 and 17:00) with microalgae (provided by SDIC Biotechnology Investment Co., Ltd., Beijing, China). Thirty minutes after feeding, residual feed and feces were removed, and 1/3 of the seawater at the same temperature was replaced. After acclimation, the oysters were fasted for 24 h to standardize the experimental conditions and to ensure that the error was small when they were weighed, and healthy and active individuals of similar size were selected for the experiments.

##### Long-Term Experiment on Suitable Growth

The natural water temperature in summer is relatively high compared to other seasons. The combined effect of thermal discharge and high summer temperatures may pose potential risks to farmed shellfish. Therefore, this experiment was based on the average summer water temperature suitable for the growth of *C. ariakensis* near a nuclear power plant. The temperature of seawater in the summer aquaculture depth is approximately 28 °C. Considering the annual and seasonal fluctuations in water temperature near nuclear power plants and the operational conditions of the plants, acclimation groups were set at 29 °C (temperature rise of 1 °C), 30 °C (temperature rise of 2 °C), and 32 °C (temperature rise of 4 °C). After the water was heated to the acclimation temperature with a heating rod at a rate of 1 °C/d, the oysters were acclimated for an additional 5 d. Other experimental conditions and acclimation procedures were the same as in the thermal tolerance experiment.

#### 2.2.2. Dynamic Thermal Tolerance Experiment

The dynamic thermal tolerance experiment involved continuously heating the water at the rate of 1 °C/d. During the experiment, three acclimation temperature groups (16 °C, 26 °C, and 30 °C) were set up, and two hundred and seventy oysters were randomly distributed in nine plastic containers (150 L), with three replicates per group and thirty oysters per replicate. The discomfort temperature was defined as the temperature at which the gap between the oyster’s valves exceeded that during normal filtration activity, and the oyster did not respond until 3 s after pressure was applied, suggesting the onset of physiological stress [11]. The critical thermal maxima (CTM) was defined as the temperature at which the oyster did not respond until 10 s of applied pressure when the oyster’s valves fully opened, indicating physiological stress before mortality [12]. The pressure was applied with a 5.0 mm diameter glass rod, which was gently touched to the middle of the oyster shell for one second.

#### 2.2.3. Static Thermal Tolerance Experiment

In the static thermal tolerance experiment, the oysters were abruptly exposed to different temperatures from an acclimated temperature, and survival and temperature tolerance ranges were recorded over a set period. The incipient lethal temperature (ILT_50_) was defined as the minimum temperature at which 50% of the test population exhibits lethal effects within a specified experimental period [13,14]. In this study, ILT_50_ refers to the lowest temperature in a series of different temperatures that results in 50% mortality among oysters after a 24-h exposure period. The static thermal tolerance experiment involved three acclimation temperatures (16 °C, 26 °C, and 30 °C), each with seven equally spaced temperatures (42–58 °C) and a control group (16 °C, 26 °C, and 30 °C, respectively). Each group had three replicates, and thirty oysters were placed in each tank (150 L). The oysters were fasted for the duration of the 24-h trail, with specialized personnel continuously monitoring oysters around the clock. The oysters’ mortality and time of death at different exposure temperatures were recorded. Mortality rates were calculated as the percentage of dead oysters within each replicate (n = 30 per tank). An oyster is considered dead when its shell is open, emits foul odors, and does not react for 30 s after the middle of the shell is lightly touched with a 5.0 mm diameter glass rod [9]. ILT_50_ was determined by linear regression between mortality rates and temperature, using interpolation to estimate the temperature corresponding to 50% mortality [11,15].

#### 2.2.4. Long-Term Experiment on Suitable Growth and Sampling

The long-term experiment on suitable growth consisted of three test groups (29 °C, 30 °C, and 32 °C) and a control group (28 °C). One hundred oysters were placed in each tank (150 L), with three replicates per group. The experiment lasted for 51 days, with one-third of the seawater replaced daily and a controlled amount of formulated feed provided regularly. Continuous aeration ensured dissolved oxygen levels above 6.0 mg/L. Water temperature, pH, and dissolved oxygen were monitored daily, and the survival of the oysters was recorded, with dead oysters removed. Before and after the experiment, 30 oysters were randomly selected from each tank to measure shell length and total weight (including the weight of the soft tissue and shell). The soft tissues of these oysters were stored in a −80 °C freezer for fat content analysis, while the digestive gland tissues were stored in liquid nitrogen for amylase activity measurement.

#### 2.2.5. Growth and Nutritional Quality of *C. ariakensis*

The wet weight of soft tissue was determined by removing the shell of the oysters and directly weighing the soft tissue.

The condition index of *C. ariakensis* was calculated using the following formula [16]:

Condition Index (%) = (Soft Tissue Wet Weight)/(Shell Weight) × 100%.

The moisture content of oysters was determined by the drying method and calculated as follows:

Moisture Content (%) = (Soft Tissue Wet Weight − Soft Tissue Dry Weight)/(Soft Tissue Wet Weight) × 100%.

The crude fat content in the soft tissue was determined using the Soxhlet extraction method [17].

#### 2.2.6. Amylase Activity in the Digestive Gland of *C. ariakensis*

Amylase activity in the digestive gland was measured using a reagent kit. Each group had three replicates and thirty digestive gland tissues per replicate. The tissue samples were homogenized (1:9, *w*/*v*) with physiological saline using a homogenizer (IKA T18 Basic), followed by centrifugation (700× *g*, 4 °C, 10 min). Afterward, the supernatant was used for analysis according to the manufacturer’s instructions (Nanjing Jiancheng Bioengineering Institute, Nanjing, China). Amylase activity was expressed as U/mgprot. One unit of amylase activity was defined as the amount of enzyme that produced 1.0 μmol of maltose per minute.

### 2.3. Statistical Analysis

The results were expressed as mean ± standard deviation (Mean ± SD). Data normality and homogeneity of variance were verified using Shapiro–Wilk and Levene’s tests, respectively. Continuous variables were analyzed via one-way ANOVA (independent variable: acclimation temperature; dependent variables: growth, nutritional quality indicators, amylase activity). For significant ANOVA results (*p* < 0.05), Duncan’s post-hoc test was selected to compare group means for its utility in identifying subgroup differences. All statistical analysis was performed using SPSS for Windows 19.0 (SPSS Inc., Chicago, IL, USA).

## 3. Results

### 3.1. Thermal Tolerance Experiment

#### 3.1.1. Discomfort Temperature and CTM

The discomfort temperatures and CTM values of *C. ariakensis* under different acclimation temperatures are shown in Table 1. As depicted, discomfort temperatures and CTM values increased with higher acclimation temperatures. Specifically, the discomfort temperatures rose from 48.6 ± 1.2 °C to 58.9 ± 3.0 °C, while the CTM values increased from 51.6 ± 1.4 °C to 61.2 ± 2.2 °C. These results indicate the oysters’ adaptive response to thermal stress.

#### 3.1.2. Incipient Lethal Temperature (ILT_50_)

The impact of temperature rise on the survival of *C. ariakensis* under different acclimation temperatures is summarized in Table 2. At the same acclimation temperature, as the test temperature increased, the mortality rate of the test organisms rapidly rose, and the time for 50% mortality shortened. Mortality rates (n = 30 per tank) were calculated as the percentage of dead oysters in each replication. Under the same test temperature (56 °C), mortality decreased, and the time for 50% mortality increased as the acclimation temperature rose.

The ILT_50_ values of *C. ariakensis* under different acclimation temperatures are shown in Table 3. As acclimation temperature increased, the ILT_50_ of *C. ariakensis* also increased, although the rate of increase gradually diminished.

### 3.2. Long-Term Experiment on Suitable Growth

#### 3.2.1. Effects of Temperature Rise on the Growth of *C. ariakensis*

After the 51-day long-term experiment on suitable growth, the increase in soft tissue wet weight for *C. ariakensis* in the 28 °C, 29 °C, 30 °C, and 32 °C test groups ranged from 2.8 to 2.9 g. Specifically, the soft tissue wet weight increased by 2.8 g in the 28 °C group, 2.6 g in the 29 °C group, 2.8 g in the 30°C group, and 2.3 g in the 32 °C group.

At the end of the experiment, compared to the initial measurements, the soft tissue wet weight in all test groups (28 °C, 29 °C, 30 °C, and 32 °C) showed a significant increase (*p* < 0.05). The soft tissue wet weight fluctuated slightly with the rise in test temperatures, but the differences between the various temperature groups were not statistically significant (*p* > 0.05) (Table 4).

#### 3.2.2. Effects of Temperature Rise on the Nutritional Quality of *C. ariakensis*

After the 51-day long-term experiment on suitable growth, the condition index of *C. ariakensis* in all test groups (28 °C, 29 °C, 30 °C, and 32 °C) showed a certain degree of increase compared to the initial measurements, with the increase ranging from 3.5% to 4.4%. Specifically, the condition index increased by 4.4% in the 28 °C group, 3.5% in the 29 °C group, 4.0% in the 30 °C group, and 4.9% in the 32 °C group.

At the end of the experiment, all test groups (28 °C, 29 °C, 30 °C, and 32 °C) showed a significant increase in the condition index compared to the initial measurements (*p* < 0.05). However, there were no significant differences in the condition index between the different temperature groups at the end of the experiment (*p* > 0.05) (Table 4).

In terms of the moisture content of soft tissue in all test groups (28 °C, 29 °C, 30 °C, and 32 °C), it showed a certain degree of increase compared to the initial measurements, with the increase ranging from 2.9% to 6.0% ww. Specifically, the moisture content increased by 5.73% ww in the 28 °C group, 6.02% ww in the 29 °C group, 3.12% ww in the 30 °C group, and 2.88% ww in the 32 °C group.

**Figure 1:**
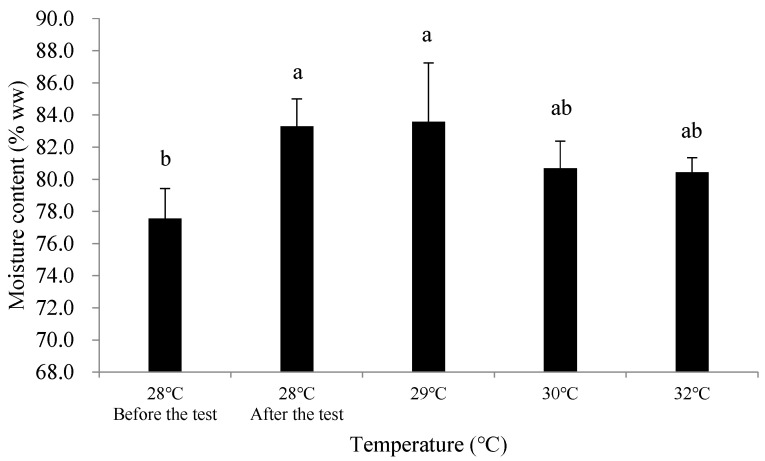
Changes in moisture content of the soft tissue of *Crassostrea* (*Magallana*) *ariakensis* in different temperature groups. Note: Values are expressed as mean ± SD (n = 30). Values with the different superscripts are significantly different (*p*  < 0.05).

At the end of the experiment, the 28 °C and 29 °C groups showed a significant increase in moisture content compared to the initial measurements (*p* < 0.05), while the 30 °C and 32 °C groups did not show significant differences (*p* > 0.05). There were no significant differences in moisture content between the different temperature groups at the end of the experiment (*p* > 0.05) (Figure 1).

After the 51-day experiment, the fat content in the soft tissue of all test groups (28 °C, 29 °C, 30 °C, and 32 °C) showed a certain degree of increase compared to the initial measurements, with the increase ranging from 0.26% to 0.43% ww. Specifically, the fat content increased by 0.40% ww in the 28 °C group, 0.43% ww in the 29 °C group, 0.26% ww in the 30 °C group, and 0.37% ww in the 32 °C group.

**Figure 2:**
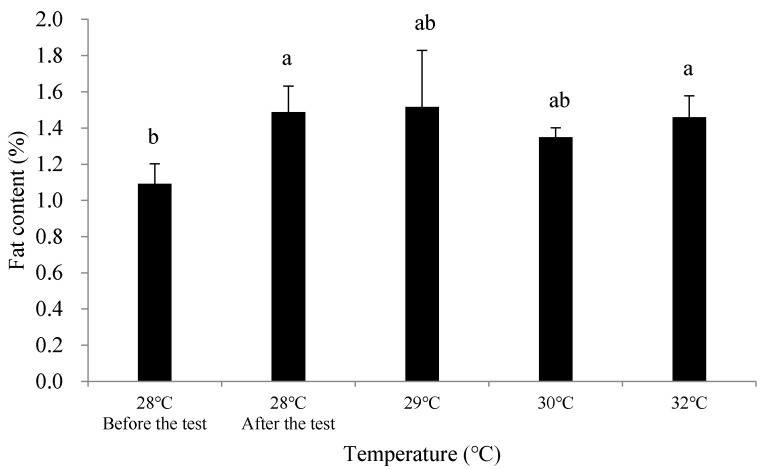
Changes in fat content in the soft tissue of *Crassostrea* (*Magallana*) *ariakensis* in different temperature groups. Note: Values are expressed as mean ± SD (n = 30). Values with the different superscripts are significantly different (*p*  < 0.05).

At the end of the experiment, the 28 °C, 29 °C, and 32 °C groups showed a significant increase in fat content compared to the initial measurements (*p* < 0.05), while the 30 °C group did not show a significant difference (*p* > 0.05). There were no significant differences in fat content between the different temperature groups at the end of the experiment (*p* > 0.05) (Figure 2).

#### 3.2.3. Effects of Temperature Rise on Amylase Activity in the Digestive Gland of *C. ariakensis*

After the 51-day long-term experiment on suitable growth, the amylase activity in the digestive gland of *C. ariakensis* decreased in all test groups (28 °C, 29 °C, 30 °C, and 32 °C) compared to the initial measurements, with the decrease ranging from 0.19 to 2.70 U/mgprot. Specifically, amylase activity decreased by 0.19 U/mgprot in the 28 °C group, 1.48 U/mgprot in the 29 °C group, 2.58 U/mgprot in the 30 °C group, and 2.70 U/mgprot in the 32 °C group.

**Figure 3:**
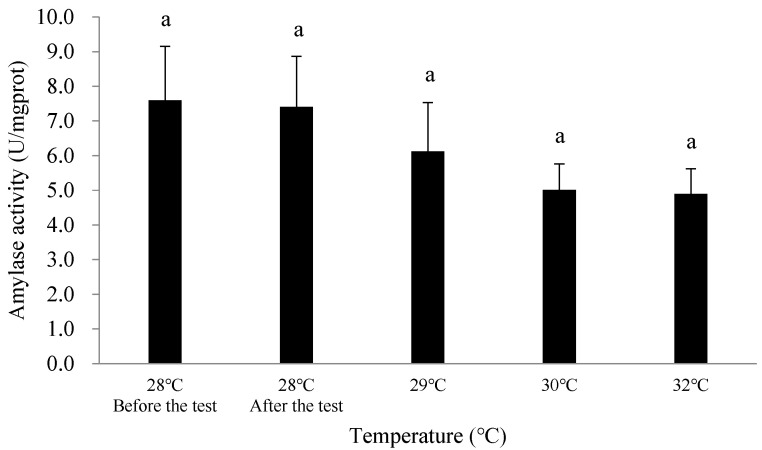
Changes in amylase activity in the digestive gland of *Crassostrea* (*Magallana*) *ariakensis* in different temperature groups. Note: Values are expressed as mean ± SD (n = 30). Values with the different superscripts are significantly different (*p*  < 0.05).

At the end of the experiment, there were no significant differences in amylase activity between the different temperature groups (*p* > 0.05). Overall, the amylase activity in the digestive gland gradually decreased as the temperature rose (Figure 3).

## 4. Discussion

### 4.1. Effects of Nuclear Plant Thermal Discharges on Water Temperature and C. ariakensis

The operation of nuclear power plants is characterized by the continuous discharge of warm water, which affects the surrounding water environment. During regular maintenance checks, the warm water discharges may be momentarily halted. This abrupt decrease in water temperature could potentially impact marine organisms such as oysters. However, the maintenance activities had very little impact on water temperature in this study. Specifically, the nuclear power plant near the study site undergoes maintenance every 18 months, during which only one unit is shut down at a time. Since the plant has multiple units, warm water continues to be discharged from the other operating units so that the seawater’s temperature fluctuation remains within ±0.5 °C. Therefore, the temperature fluctuation during maintenance activities has a negligible effect on *C. ariakensis*.

### 4.2. Effects of Temperature Rise on the Heat Tolerance of C. ariakensis

Heat tolerance levels reflect an organism’s capacity to endure in a specific thermal environment. As poikilotherms, bivalves have limited ability to regulate their body temperature and are thus susceptible to biotic and abiotic stressors in their environment, restricting their survival to a tolerable temperature range [18].

In this experiment, there was a relationship between temperature and the mortality rate of *C. ariakensis*, with mortality rates increasing as the temperature rose (Table 2). This trend is consistent with previous studies on various bivalve species such as *Lutraria siebaldii* [19], *Patinopecten yessoensis* [20], *Argopectenirradians concentricus* [21], *Ruditapes philippinarum* [22], *Modiolus barbatus* [23], and *Meretrix lyrate* [24], suggesting a general pattern in bivalve mortality as a function of temperature. Previous research suggests that oxidative stress mediated by temperature plays a crucial role in determining the heat tolerance limits of bivalves [25]. As the temperature increases, so do respiration and metabolic rates, leading to an increase in reactive oxygen species (ROS) under stress conditions [26,27]. While ROS are part of the body’s defense mechanism against pathogens, excessive ROS disrupts the balance with the antioxidant system, resulting in toxic effects by reacting with DNA, proteins, and lipids [26,28]. Additionally, high temperatures can cause negative changes in water quality, such as reduced dissolved oxygen levels, decreased transparency, and intensified mineralization [29]. The combined stress of high temperatures and adverse environmental factors damages the functions of *C. ariakensis*, ultimately leading to mortality.

Within a certain range of temperature rise, organisms can survive by adjusting their physiological functions. However, this adjustment has its limits. In this experiment, as the test temperature increased at each acclimation temperature, the mortality rate of *C. ariakensis* increased rapidly, and the time to 50% mortality decreased. Typically, temperature increases activate metabolic enzymes, ion channels, mitochondria, and other crucial biochemical processes to compensate for temperature stress [30,31]. However, when the exposure temperature exceeds the organism’s maximum adjustment limit, the physiological compensation mechanism weakens and eventually collapses, leading to shorter survival times.

In this experiment, the ILT50 of *C. ariakensis* increased as the acclimation temperature rose, indicating that acclimation can alter the heat tolerance of *C. ariakensis*. The metabolic adjustments induced by gradual temperature changes, known as “thermal acclimation”, can enhance an organism’s heat tolerance after a period of adaptation below the extreme temperature. This phenomenon has been validated in various species, including bivalves [9,32]. Under the same acclimation temperature of 16 °C, *C. ariakensis* exhibited a higher CTM value compared to species like *Tegillarca granosa*, *Crassostrea gigas*, *Sinonovacula constricta*, and *Mytilus galloprovincialis* when compared with the results of the thermal tolerance experiment in other shellfish species (Table 5). This suggests that *C. ariakensis* exhibits relatively high plasticity in heat tolerance and can be cultured in areas with mild thermal discharges. In other words, the findings of this experiment suggest that oyster farming activities can be sustained in seawater where the temperature has increased by up to 4 °C due to thermal discharges.

### 4.3. Effects of Temperature Rise on the Nutritional Quality of C. ariakensis

Nutritional quality indicators, such as the condition index, are important determinants of the market value of *C. ariakensis*. In this experiment, there were no significant differences in the condition index between the 28 °C, 29 °C, 30 °C, and 32 °C groups, and all were significantly higher than before the experiment (Table 4). This suggests that the temperature rise had a minimal impact on the condition index of *C. ariakensis*.

The moisture content of *C. ariakensis*’s soft tissue is an important indicator affecting its taste. In this experiment, the moisture content of soft tissue in the 30 °C and 32 °C groups was lower than that in the 28 °C and 29 °C groups (Figure 1), indicating that the temperature rise started to affect the moisture content at 30 °C.

Fat content is a crucial indicator of the nutritional quality of bivalves. In bivalves, lipids are mainly stored in the hepatopancreas, with a distinct seasonal pattern of synthesis, storage, and utilization—lipids generally accumulate in summer and are used for metabolism in winter [34]. In this experiment, the soft tissue’s fat content of *C. ariakensis* in the 28 °C, 29 °C, 30 °C, and 32 °C groups all increased compared to the pre-experimental group. The fat content in the 30 °C and 32 °C groups was lower than that in the 28 °C and 29 °C groups (Figure 2), suggesting that the temperature rise at 30 °C and 32 °C affected the fat content in the soft tissue. This may be due to the enhanced metabolic rate of *C. ariakensis* under the temperature rise, leading to metabolic imbalance and higher energy demands, with fat being converted into usable energy under certain conditions [35]. Furthermore, because bivalves have restricted mobility in their native habitat, lipids are essential for their growth, development, and reproduction. Therefore, temperature rise may have a greater impact on the long-term survival and reproduction of bivalves in natural environments.

### 4.4. Effects of Temperature Rise on the Amylase Activity in the Digestive Gland of C. ariakensis

Temperature is a dominant factor controlling metabolic processes in organisms, affecting the chemical reactions involved in enzyme synthesis, which in turn determine the general state of the organism, including growth, development, and reproduction [4]. Digestive enzymes, such as amylase, are key indicators of an organism’s digestive capacity and mechanism, catalyzing the hydrolysis of starch and glycogen. Amylase is a temperature-dependent enzyme, and temperature plays a crucial role in its biochemical activity and stability, ultimately affecting the metabolic functions of the organism [36]. After the 51-day long-term experiment on suitable growth, the amylase activity in the digestive gland of *C. ariakensis* gradually decreased with increasing temperature across different test groups; however, there was no significant difference between the before and end of the experiment (Figure 3). This indicates that a certain degree of temperature rise over a limited time does not significantly affect the amylase activity in the digestive gland of *C. ariakensis*. This trend is consistent with the changes in soft tissue wet weight observed in the different temperature test groups. However, the downward trend in amylase activity with rising temperature is consistent with results observed in other bivalves such as *Mytilus coruscus* [37] and *Hyriopsis bialatus* [38]. While reduced amylase activity did not immediately affect growth, sustained inhibition could impair carbohydrate digestion and affect energy allocation [39]. Therefore, more research is required to quantify feeding rates under thermal stress and to examine the long-term effects of warming discharges on *C. ariakensis* in its natural summer environment.

## 5. Conclusions

In this study, we simulated the average water temperature in different seasons near the coastal nuclear power plants and observed that the CTM value and the ILT_50_ of *C. ariakensis* increased as the acclimation temperature rose. This finding indicates that *C. ariakensis* has some degree of thermal tolerance, aligning with our initial hypothesis of the “thermal acclimation” mechanism in this species. Over the 51-day long-term experiment on suitable growth, no statistically significant impacts were observed on the soft tissue wet weight, condition index, moisture content, and fat content of *C. ariakensis*, indicating short-term resilience to moderate warming. However, the amylase activity in the digestive gland presented a downward trend, and lipid reserves were reduced in higher temperature groups, suggesting latent metabolic costs that could compromise long-term energy allocation for survival. These findings highlight that while *C. ariakensis* shows short-term thermal tolerance, chronic exposure to elevated temperatures may gradually weaken physiological resilience. Based on the results of this experiment, oyster farming activities can be sustained in seawater where the temperature has increased by up to 4°C due to thermal discharges. To safeguard aquaculture productivity, we propose that the fluctuations of digestive enzyme activity and lipid profiles should be monitored during peak summer temperatures. In summary, our study provides a basic understanding of the thermal tolerance of *C. ariakensis* and theoretical support for strategic planning in marine aquaculture.

## Figures and Tables

**Table 1:** Discomfort temperature and critical thermal maxima (CTM) values of *Crassostrea* (*Magallana*) *ariakensis* at different acclimation temperatures.

Acclimation Temperature (°C)	16	26	30
Discomfort temperature (°C)	48.6 ± 1.2	57.8 ± 2.9	58.9 ± 3.0
CTM (°C)	51.6 ± 1.4	60.0 ± 1.6	61.2 ± 2.2

**Table 2:** Effect of temperature rise on the survival of *Crassostrea* (*Magallana*) *ariakensis* at different acclimation temperatures.

Acclimation Temperature (°C)	Indicators	Exposure Temperature (°C)						
		42	43	44	45	46	47	48
16	Mortality rate (%)	0	0	7.8 ± 1.9	38.9 ± 5.1	58.9 ± 8.8	82.2 ± 12.0	100.0 ± 0.0
	Time for 50% mortality (min)	-	-	-	-	1045 ± 100.1	527 ± 52.9	125 ± 20.1
**Acclimation Temperature (°C)**	**Indicators**	**Exposure Temperature (°C)**						
		**50**	**51**	**52**	**53**	**54**	**55**	**56**
26	Mortality rate (%)	0	5.6 ± 0.5	13.3 ± 1.0	30.0 ± 1.3	53.3 ± 1.7	74.4 ± 2.0	100.0 ± 0.0
	Time for 50% mortality (min)	-	-	-	-	1231 ± 110.0	746 ± 88.1	357 ± 20.1
**Acclimation Temperature (°C)**	**Indicators**	**Exposure Temperature (°C)**						
		**52**	**53**	**54**	**55**	**56**	**57**	**58**
30	Mortality rate (%)	0	0	4.4 ± 0.7	16.7 ± 0.7	52.2 ± 1.8	74.4 ± 2.6	100 ± 0.0
	Time for 50% mortality (min)	-	-	-	-	1440 ± 156.5	932 ± 46.3	181 ± 10.6

Note: “-” means no data.

**Table 3:** Incipient lethal temperature (ILT_50_) of *Crassostrea* (*Magallana*) *ariakensis* at different acclimation temperatures.

Acclimation Temperature (°C)	16	26	30
ILT_50_ (°C)	45.61	53.71	55.90
95% Confidence Interval (°C)	45.43–45.82	53.50–53.94	55.70–56.13

**Table 4:** Changes in soft tissue wet weight and condition index of *Crassostrea* (*Magallana*) *ariakensis* in different temperature groups.

Indicators	Temperature (°C)				
	28 (Before the Test)	28 (After the Test)	29	30	31
Soft Tissue Wet Weight (g)	4.1 ± 1.8 ^b^	6.9 ± 2.2 ^a^	6.7 ± 2.2 ^a^	6.9 ± 1.7 ^a^	7.0 ± 1.3 ^a^
Condition Index (%)	7.3 ± 3.6 ^c^	11.7 ± 1.2 ^a^	10.8 ± 1.8 ^a^	11.3 ± 1.9 ^a^	12.2 ± 1.2 ^a^

Note: Values are expressed as mean ± SD (n = 30). Values with the different superscripts are significantly different (*p* < 0.05).

**Table 5:** CTM values of different shellfish species.

Species	Temperature (°C)	CTM (°C)	References
*C. ariakensis*	16	51.6 ± 1.4	This paper
*Tegillarca granosa*	16	39.3	[33]
*Crassostrea gigas*	16	38.8	[33]
*Sinonovacula constricta*	16	38.3	[33]
*Mytilus galloprovincialis*	16	32.2	[33]

## Data Availability

The data referenced in this article is included within the text.
